# Enzyme Localization Can Drastically Affect Signal Amplification in Signal Transduction Pathways

**DOI:** 10.1371/journal.pcbi.0030195

**Published:** 2007-10-12

**Authors:** Siebe B van Albada, Pieter Rein ten Wolde

**Affiliations:** FOM Institute for Atomic and Molecular Physics, Amsterdam, The Netherlands; University of Illinois, United States of America

## Abstract

Push–pull networks are ubiquitous in signal transduction pathways in both prokaryotic and eukaryotic cells. They allow cells to strongly amplify signals via the mechanism of zero-order ultrasensitivity. In a push–pull network, two antagonistic enzymes control the activity of a protein by covalent modification. These enzymes are often uniformly distributed in the cytoplasm. They can, however, also be colocalized in space; for instance, near the pole of the cell. Moreover, it is increasingly recognized that these enzymes can also be spatially separated, leading to gradients of the active form of the messenger protein. Here, we investigate the consequences of the spatial distributions of the enzymes for the amplification properties of push–pull networks. Our calculations reveal that enzyme localization by itself can have a dramatic effect on the gain. The gain is maximized when the two enzymes are either uniformly distributed or colocalized in one region in the cell. Depending on the diffusion constants, however, the sharpness of the response can be strongly reduced when the enzymes are spatially separated. We discuss how our predictions could be tested experimentally.

## Author Summary

Living cells continually have to respond to a changing environment. To this end, they do not only have to detect environmental signals, but also to amplify them. In living cells, signals are often amplified in so-called push-pull networks. In a push–pull network, two enzymes control the activity of a protein in an antagonistic manner. A well-known example is a network in which a kinase phosphorylates a messenger protein, while a phosphatase dephosphorylates the same protein. While it has long been assumed that the enzymes are uniformly distributed in the cytoplasm, it is increasingly becoming clear that in many systems one or both of the enzymes are localized in space, for instance near the cell pole. If the enzymes are spatially separated, then spatial gradients of the messenger protein can form, and recently a number of these protein gradients have been observed experimentally. We study by numerical calculations how the amplification properties of push–pull networks depend upon the spatial distribution of the enzymes. We find that the gain is maximized when the enzymes are either uniformly distributed or colocalized in space. Depending upon the diffusion constants, however, the sharpness of the response can be strongly reduced when the enzymes are spatially separated.

## Introduction

**Figure 1:**
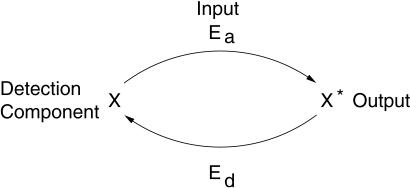
A Push–Pull Network Two enzymes, E_a_ and E_d_, covalently (de)modify the components X and X^*^, respectively. The activating enzyme E_a_ provides the input signal, the unmodified component X is the detection component, and the modified component X^*^ provides the output signal.

Living cells are information processing machines. To process information reliably, signals often need to be amplified. To this end, cells can employ a variety of amplification mechanisms. Signals can be amplified via positive feedback, cooperative binding of signaling molecules to receptors, or interactions between receptor molecules [1]. Another principal mechanism for signal amplification is zero-order ultrasensitivity [2,3]. This mechanism operates in so-called push–pull networks, which are omnipresent in both prokaryotes and eukaryotes. In a push–pull network, two enzymes covalently modify a component in an antagonistic manner (see Figure 1). One well-known example is a network in which a kinase phosphorylates a component, and a phosphatase dephosphorylates the same component. If both enzymes operate near saturation, then the modification reactions become zero order, which means that the reaction rates become insensitive to the substrate concentrations. Under these conditions, a small change in the concentration of one of the two enzymes (the input signal), will lead to a large change in the concentration of the modified protein (the output signal) [2,3]. The amplification properties of push–pull networks have been analyzed in detail [2–8]. In these studies, however, it is assumed that the antagonistic enzymes are uniformly distributed in space. Yet, it is increasingly recognized that in many systems one or both of the two antagonistic enzymes are localized in space, for instance at the cell pole. Here, we address the question how the spatial distribution of the antagonistic enzymes affects the amplification properties of push–pull networks.

If the two antagonistic enzymes are separated in space, then gradients of the messenger protein can form [9–13]. Recently, a number of protein gradients have been observed experimentally in both prokaryotic and eukaryotic cells. For example, in Escherichia coli cells, the kinase CheA and the phosphatase CheZ control the phosphorylation level of the messenger CheY, which transmits the chemotactic signal from the receptor cluster to the flagellar motors. In wild-type cells, the kinase and the phosphatase are both localized at the receptor cluster [14], and, as a result, the steady-state concentration profile of CheY is uniform [10]. However, in E. coli mutants, where the phosphatase is distributed in the cytoplasm, gradients of CheY have recently been observed [10]. Other examples of protein gradients include Caulobacter, in which MipZ gradients guide chromosome segregation and division site selection [15]. In eukaryotic cells, gradients of Ran, Stathmin, and HURP proteins aid in the formation of the mitotic spindle by providing directional cues for microtubule growth [16–19]. Moreover, in eukaryotic cells, the kinases in the mitogen-activated protein kinase (MAPK) cascade often bind to scaffold proteins, while the phosphatases are distributed in the cytoplasm [20]. This will lead to concentration gradients of the activated kinases, which can become particularly important if the scaffolds are located near the membrane.

In this study, we compare the amplification properties of a canonical push–pull network, where all components are uniformly distributed in space, with those of a network in which the enzyme that provides the input signal is localized at one end of the cell, while all the other components can freely diffuse through the cell. In the latter case, the concentration profile of the messenger—the output signal—is non-uniform. Previous studies have focused on the time-dependent concentration profiles of the messenger [9,12,13] and on the “control” of diffusion over protein fluxes [21] in similar systems. Here, we examine the effect of the spatial distribution of the enzymes on the amplification properties of push–pull networks. To this end, we compute for both systems the steady-state input–output relations. Our analysis reveals that the spatial distribution of the enzymes can have a dramatic effect on the capacity of push–pull networks to amplify input signals: the maximum gain of the network in which one enzyme is localized at one end of the cell, while the other is not, can be much lower than that of the network in which the components are uniformly distributed in space. Importantly, this effect occurs over a range of diffusion constants, protein concentrations, and enzymatic activities that is typical for living cells.

In the next section, we introduce the push–pull network. In the Results section, we first present the input–output relations for both networks. We show that the gain can be much reduced when the enzymes are spatially separated, and demonstrate that the magnitude of this effect depends upon the diffusion constants of the diffusing components. To elucidate the dose–response curves, we discuss in the subsequent sections the spatial concentration profiles in both the low and high activation limits. This analysis reveals that the maximum gain in the non-uniform system is reduced, because the response of the network depends on the position in the cell. Interestingly, the calculations also show that separating the enzymes in space does not only attenuate strong signals by limiting the maximum response, but can also enhance the propagation of weak signals.

## Methods

### The Push–Pull Network

A push–pull network consists of two Michaelis-Menten reactions (see also Figure 1):

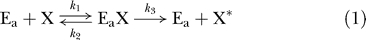


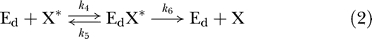



Here, E_a_ is the activating enzyme that provides the input signal, and E_d_ is the deactivating enzyme. The substrate X is the unmodified messenger that serves as the detection component and X^*^ is the modified messenger that provides the output signal; E_a_X denotes the activating enzyme bound to its substrate X, and E_d_X^*^ is the deactivating enzyme bound to its substrate X^*^.

If all the components are uniformly distributed in space, then the chemical rate equations that correspond to this network are:

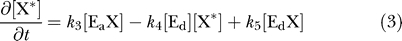


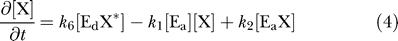


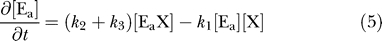


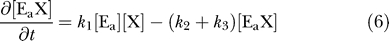


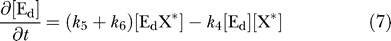


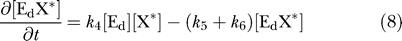



Here, […] denotes the concentrations of the species. The steady-state input–output curve of this network can be obtained analytically [2].

We will compare the behavior of this network with that of a network in which the activating enzyme E_a_ is located at one pole of the cell, while the other components can freely diffuse in the cytoplasm. The cell is assumed to be cylindrically symmetric. Since we are interested in the mean concentration profiles, it is meaningful to integrate out the lateral dimensions *y* and *z*. We thus consider a simplified 1-D model, with concentrations as a function of *x* only. This leads to the following reaction–diffusion equations:

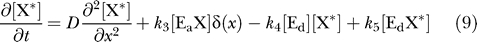


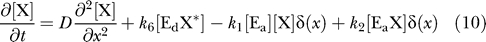


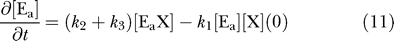


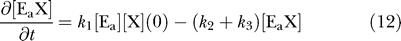


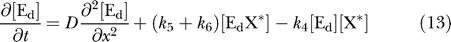


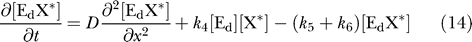



The components E_a_ and E_a_X are localized in the membrane at one end of the cell; the unit of their concentrations is the number of molecules per area. The other components diffuse in the cell. Their concentrations, which are in units of number of molecules per volume, depend on the position *x* in the cell, where *x* measures the distance from the pole at which E_a_ and E_a_X are localized; only in Equations 11 and 12 is the *x* dependence explicitly indicated to emphasize that the E_a_–X association rate depends on the concentration of X at contact. Zero-flux boundary conditions are imposed at both cell ends. The steady-state input–output relations of the network described by Equations 9–14 were obtained numerically by discretizing the system on a (1-D) grid and propagating Equations 9–14 in space and time until steady state was reached.

We consider a cell with the typical dimensions of an E. coli cell: the length of the cell, *L*, is thus on the order of 3 *μ*m [10]. We assume the same diffusion constants for all the components that can diffuse in the cytoplasm. This is for reasons of simplicity; it is not essential for the main conclusions of our work. To focus on the effect of enzyme localization on the input–output relation, we assume for both networks that *k*_1_ = *k*_4_, *k*_2_ = *k*_5_, *k*_3_ = *k*_6_; the Michaelis-Menten constants for the modification and demodification reactions are thus the same: *K*_M,a_ ≡ (*k*_2_ + *k*_3_) / *k*_1_ = *K*_M,d_ ≡ (*k*_5_ + *k*_6_) / *k*_4_. To compare the two networks on equal footing, the total concentration of activating enzyme, [E_a_]_T_ ≡ [E_a_] + [E_a_X], was chosen such that 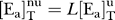 , where 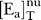 is the concentration (per unit area) in the non-uniform system and 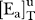 is the concentration (per unit volume) in the spatially uniform network. This choice ensures that the total number of activating enzyme molecules in the whole cell is the same for both systems. In what follows, we will report 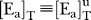 .

In the calculations, we vary the concentration of the activating enzyme, E_a_, which is the input signal. The total concentration of the deactivating enzyme, E_d_, is kept constant at [E_d_]_T_ = 0.5 *μ*M; the rate constants are fixed at *k*_1_ = *k*_4_ = 10^8^ M^−1^s^−1^, *k*_2_ = *k*_5_ = 25 s^−1^, *k*_3_ = *k*_6_ = 25 s^−1^, corresponding to Michaelis-Menten constants of *K*_M_ = *K*_M,a_ = *K*_M,d_ = 0.5 *μ*M. We will study extensively the effect of changing the diffusion constant *D* and the total substrate concentration [S]_T_ ≡ [X]_T_ + [X^*^]_T_, where [X]_T_ ≡ [X] + [E_a_X] / *L* is the total concentration of X and [X^*^]_T_ ≡ [X^*^] + [E_d_X^*^] is the total concentration of X^*^. Their base-line parameters, however, are: *D* = 10 *μ*m^2^s^−1^ and [S]_T_ = 20 *μ*M. The magnitude of the diffusion constant [22], as well as the values of the Michaelis-Menten constants, enzyme concentrations, and substrate concentrations, are typical for prokaryotic [23] and eukaryotic cells [4].

## Results

### The Input–Output Relation

**Figure 2:**
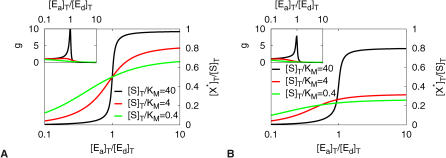
The Effect of Enzyme Localization on the Response of a Push–Pull Network The input–output relation of the push–pull network shown in Figure 1 is plotted for different values of the total substrate concentration [S]_T_, for the case in which all components are uniformly distributed in space (A) and for the case in which the activating enzyme is located at one end of the cell, while the other components can diffuse freely through the cell (B). Here, 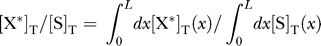 . In (A) and (B), [E_d_]_T_ = 0.5 *μ*M, *K*_M,a_ = *K*_M,d_ = 0.5 *μ*M, and *k*_3_ = *k*_6_ = 25s^−1^. In (B), the diffusion constant is *D* = 10 *μ*m^2^ s^−1^. The inset shows the logarithmic gain *g* ≡ ∂ln[X^*^]_T_ / ∂ln[E_a_]_T_. It is seen that the sharpness of the response increases markedly with increasing substrate concentration when all the components are uniformly distributed in space (A), but much less so when the activating enzyme E_a_ is located at one pole of the cell, while the deactivating enzyme E_a_ is distributed in the cytoplasm. When both enzymes E_a_ and E_d_ are located at one pole, the steady-state dose–response curve is identical to that in (A).

Goldbeter and Koshland showed that if the antagonistic enzymes in a push–pull network operate near saturation (see Figure 1), a small change in the concentration of the activating enzyme E_a_ can lead to a large change in the output, the modified messenger X^*^ [2]. The enzymes become more saturated with substrate when either the Michaelis-Menten constants *K*_M,a_ and *K*_M,d_ decrease, or the total substrate concentration [S]_T_ = [X] + [E_a_X] / *L* + [E_d_X^*^] + [X^*^] increases. Figure 2A shows the steady-state input–output relation for a push–pull network in which all the components are uniformly distributed in space, for different substrate concentrations. It is seen that as the substrate concentration is increased, the sharpness of the response is drastically enhanced. This is the hallmark of the mechanism of zero-order ultrasensitivity.

In many systems, such as the bacterial chemotaxis network of E. coli [10], the two antagonistic enzymes are colocalized at the same pole, while the detection component X and the messenger X^*^ can diffuse through the cytoplasm. While the time-dependent response curves of such a network will differ from those of the two networks considered here, the steady-state dose–response curves will be identical to those of a network in which all the components are homogeneously distributed in the cytoplasm. The response curves shown in Figure 2A thus also pertain to push–pull networks in which the two enzymes are colocalized at one end of the cell, while their substrates freely diffuse in the cytoplasm. Indeed, also in these networks the mechanism of zero-order ultrasensitivity can strongly amplify input signals.

#### Spatially separating the enzymes reduces the gain.

Figure 2B shows the dose–response curves for a push–pull network in which the activating enzyme E_a_ is localized at one pole of the cell, while the other components diffuse in the cytoplasm. Three points are worthy of note. The first is that the maximum output signal, the concentration of the messenger X^*^, is much lower than that of the corresponding network in which all components are uniformly distributed in space (see Figure 2). In fact, while in the spatially uniform network, the fraction of modified substrate, [X^*^]_T_ / [S]_T_, always approaches unity if [E_a_]_T_ / [E_d_]_T_ becomes large; in the non-uniform network the fraction of modified substrate saturates to a lower level: even when the concentration of activating enzyme is much higher than that of the deactivating enzyme, not all substrate X is converted into X^*^. The second point to note is that as the total substrate concentration decreases, the inflection point of the dose–response curve shifts to lower values of [E_a_]_T_ / [E_d_]_T_. The last, and perhaps most important, point to note is that the *sharpness* of the response of the network is much weaker than that of the network in which the enzymes are either colocalized or uniformly distributed in space. The insets of Figure 2 show the logarithmic gain, g ≡ ∂ln[X^*^]_T_ / ∂ln[E_a_]_T_, as a function of [E_a_]_T_ / [E_d_]_T_ for both networks ([E_d_]_T_ is kept constant). It is seen that for both low [E_a_]_T_ / [E_d_]_T_ and high [E_a_]_T_ / [E_d_]_T_ the gain is small and fairly similar for both networks, while for the symmetric networks considered here, at [E_a_]_T_ ≈ [E_d_]_T_ the gain is maximal, but smaller for the network in which the enzymes are spatially separated. Hence, spatially separating the two antagonistic enzymes reduces the maximum gain of a push–pull network.

#### The dose–response curves strongly depend on the diffusion constants.

**Figure 3:**
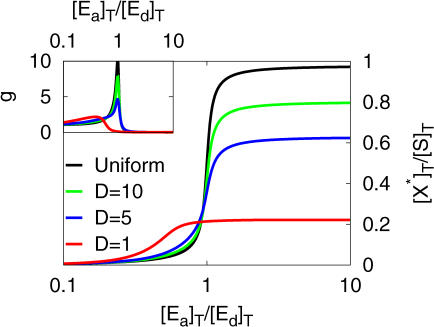
Effect of the Diffusion Coefficient on the Response The input–output relation of a network in which the activating enzyme is located at one pole, while the other components can freely diffuse in the cytoplasm, is plotted for different values of the diffusion constant *D* (in *μ*m^2^s^−1^) of the cytoplasmic components. The inset shows the logarithmic gain *g* ≡ ∂ln[X^*^]_T_ / ∂ln[E_a_]_T_. It is seen that the gain of the push–pull network strongly increases with increasing diffusion constant. If *D* → ∞, the dose–response curve approaches that of the push–pull network in which the components are uniformly distributed in space (and that of the network in which the enzymes are colocalized). The total substrate concentration is [S]_T_ = 20 *μ*M, the total concentration of the deactivating enzyme is [E_d_]_T_ = 0.5 *μ*M, the Michaelis-Menten constants are *K*_M,a_ = *K*_M,d_ = 0.5 *μ*M, and the catalytic rate constants are *k*_3_ = *k*_6_ = 25s^−1^.

The extent to which the spatial separation of the opposing enzymes can change the response of the network depends on the magnitude of the diffusion constant of the components. This is illustrated in Figure 3. Figure 3 shows the input–output relation of a push–pull network where the activating enzyme is located at one end of the cell, while the other components diffuse freely in the cytoplasm, for different values of the diffusion constant. This network is in the zero-order regime: the total substrate concentration is large compared with the concentrations of the enzymes and the Michaelis-Menten constants. Yet, for low values of the diffusion constants, the response is rather weak. As the diffusion constant increases, however, the sharpness of the response markedly increases. For *D* → ∞, the input–output relation approaches that of a push–pull network in which all components are either uniformly distributed in space, or colocalized in one region of the cell.

#### Spatially separating the enzymes attenuates the propagation of strong signals, but can enhance the transmission of weak signals.

Figure 3 shows that in a zero-order network in which only the activating enzyme is localized at one pole of the cell, the concentration of X^*^ decreases with decreasing diffusion constant when [E_a_]_T_ > [E_d_]_T_, but *increases* with decreasing diffusion constant when [E_a_]_T_ < [E_d_]_T_. This means that when the input signal is strong (high kinase activity), the response of a network in which the enzymes are spatially separated is weaker than that of a network in which the enzymes are either uniformly distributed or colocalized in space; conversely, when the input signal is weak (low kinase activity), the spatially non-uniform network can respond more strongly than a uniform network. Spatially separating the antagonistic enzymes will thus attenuate strong input signals, but can also amplify weak input signals.

### Mechanism: Concentration Gradients

To explain the effect of enzyme localization on the amplification properties of push–pull networks, it is instructive to consider the effect of diffusion on the input–output relation: in the limit that *D* → ∞, the response of the network in which the activating enzyme is located at the pole, while the other is distributed in the cytoplasm, approaches that of a network in which the enzymes are either uniformly distributed in space or colocalized at the pole. The effect of diffusion on the response curves can be understood by considering the effects of diffusion on the spatial concentration profiles.

**Figure 4:**
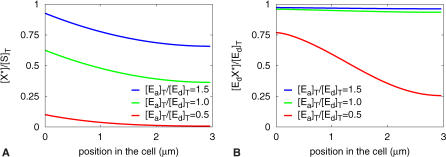
Concentration Profiles of a Spatially Non-Uniform Push–Pull Network The concentration profiles of X^*^ (A) and E_d_X^*^ (B) in a push–pull network in which the activating enzyme is located at one pole of the cell, while the other components are distributed in the cytoplasm, for three different concentrations of the activating enzyme. For all curves, [S]_T_ = 20 *μ*M, [E_d_]_T_ = 0.5 *μ*M, *K*_M,a_ = *K*_M,d_ = 0.5 *μ*M, *k*_3_ = *k*_6_ = 25s^−1^, and *D* = 10 *μ*m^2^ s^−1^.

In a push–pull network where the antagonistic enzymes are either uniformly distributed or colocalized in space, the steady-state spatial concentration profiles of the freely diffusing components are uniform across the cell. In a push–pull network where the two antagonistic enzymes are spatially separated, concentration gradients of the freely diffusing components can form. Figure 4 shows for a zero-order network in which the activating enzyme is located at one pole of the cell, while the other is not, the concentration profiles of X^*^ and E_d_X^*^, for three different (total) concentrations of the activating enzyme E_a_, [E_a_]_T_. The concentrations of X^*^ and E_d_X^*^ are highest near the pole where X is activated, and decay in the cytoplasm where X^*^ is deactivated. Moreover, the concentration profiles increase as [E_a_]_T_ increases. These gradients impose fundamental limits on the maximum gain of the system.

To clarify the effect of diffusion on the concentration profiles and the input–output relations, it is instructive to recall that, in general, the spatio–temporal evolution of [X^*^] is given by the interplay of activation, deactivation, and diffusion of X^*^:

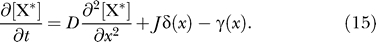



Here, *J* denotes the influx of X^*^ into the system, while *γ* denotes the deactivation rate of X at position *x*. If the formation of the enzyme–substrate complexes is fast (see Text S1), then *J* and *γ* are given by

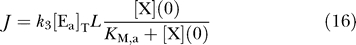


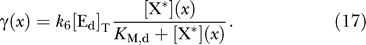



Here, [E_a_]_T_ ≡ [E_a_] + [E_a_X] and [E_d_]_T_ ≡ [E_d_] + [E_d_X^*^] are the total concentrations of E_a_ and E_d_, respectively. Combining Equation 13 with Equation 14 reveals that the total concentration profile of E_d_, [E_d_]_T_(*x*), is constant in space if, as assumed here, the diffusion constants of the enzyme E_d_, and that of the enzyme bound to its substrate, E_d_X^*^, are the same. The synthesis rate of X^*^ depends upon the concentration of X at contact and hence upon the concentration of X^*^ at contact; similarly, the deactivation rate of X^*^ at position *x* depends upon the concentration of X^*^ at *x*. This is important to note, because, as we discuss below, the dose–response curves are determined by the sensitivities of the influx *J* and the deactivation rate *γ* to changes in the substrate concentration. We will now first discuss the input–output relations of zero-order push–pull networks, and then briefly the response curves of push–pull networks that are in the linear regime.

### Push–Pull Networks in the Zero-Order Regime

**Figure 5:**
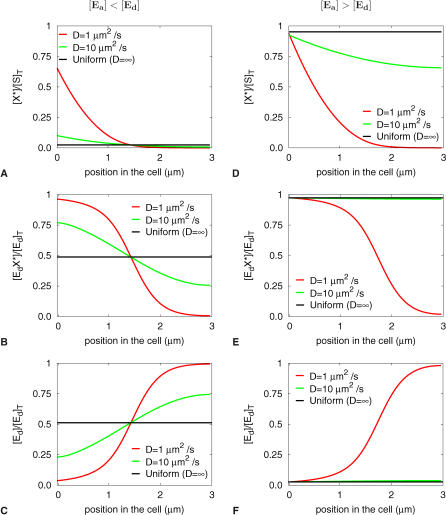
Effect of Diffusion on the Concentration Profiles: Weak versus Strong Activation Profiles of [X^*^] (A,D), [E_d_X^*^] (B,E), and [E_d_] (C,F). (A–C) Low concentration of activating enzyme, [E_a_]_T_ = 0.5[E_d_]_T_. (D–F) High activating enzyme concentration, [E_a_]_T_ = 1.5[E_d_]_T_. For the other parameter values, see Figure 4.

**Figure 6:**
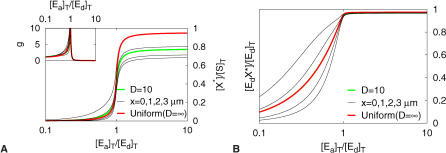
Response Curves at Different Positions in the Cell Dose–response curves of the push–pull network in which the activating enzyme is localized at one pole of the cell, while the other components diffuse in the cytoplasm, for different positions in the cell (*x* = 0 corresponds to the black left most curve, while *x* = 3 *μ*m corresponds to the black right most curve). Profiles of [X^*^] (A) and profiles of [E_d_X^*^] (B); note that the response becomes sharper farther away from the pole. The green curves correspond to the average or integrated response of the non-uniform system, while the red curves correspond to the uniform system. The inset shows the logarithmic gain *g* ≡ ∂ln[X^*^] / ∂ln[E_a_]_T_ at the respective positions in the cell (*x* = 0,1,2,3, *μ*m). For the parameter values, see Figure 4.

Figures 4–6 show the concentration profiles and dose–response curves of push–pull networks that are in the zero-order regime. We now discuss the limits of weak and strong activation separately.

### Weak Activation

We first consider the regime in which the concentration of the activating enzyme is lower than that of the deactivating enzyme, corresponding to Figure 5A–5C. In the limit that [E_a_]_T_ ≪ [E_d_]_T_, [X] will be large and [X^*^] will be small. As a consequence, E_a_ is saturated with its substrate X, while E_d_ is not saturated with its substrate X^*^. Because E_a_ is saturated, the influx of X^*^ into the system is constant (i.e., independent of [X] and [X^*^]) and given by *J* = *k*_3_[E_a_]_T_ *L* (see Equation 16). Because E_d_ is unsaturated, the deactivation rate *γ* is proportional to [X^*^]: *γ*(*x*) = *μ*[X^*^](*x*), with *μ* = *k*_6_ / *K*_M,d_[E_d_]_T_ (see Equation 17). This means that in this regime the deactivation rate per particle is constant.

With the influx *J* being constant and the deactivation rate *γ* being proportional to [X^*^], Equation 15 can be solved analytically (see Text S1). Defining the characteristic decay length of X^*^ to be 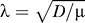 , then, if *L* ≫ *λ*, the solution is

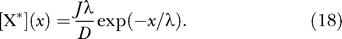



Equation 18 reveals that when *D* increases, the profile decays more slowly, and the concentration of X^*^ at contact decreases. When *D* increases, the X^*^ molecules diffuse away from the pole more rapidly. Because in the regime considered here, namely [E_a_]_T_ ≪ [E_d_]_T_, the influx of X^*^ is constant and independent of *D*, the concentration of X^*^ close to the pole will decrease when the molecules diffuse away more rapidly, while the concentration farther away will increase. In fact, in this limit, the total concentration of X^*^, [X^*^]^cell^, is independent of the diffusion constant; this can be verified by integrating Equation 18 over the whole cell, which yields [X^*^]^cell^ = *J* / *μ*. The fact that the total concentration of [X^*^] is independent of the diffusion constant, means that the response of the network does not depend upon the spatial distribution of the enzymes.

When [E_a_]_T_ increases, [X^*^] increases and [X] decreases. As a result, E_a_ becomes less saturated, while E_d_ becomes saturated. Hence, the influx *J* will at some point become sensitive to X, while the deactivation rate *γ* will no longer be proportional to [X^*^]. However, in the zero-order regime considered here, the total substrate concentration [S]_T_ is large compared with the enzyme concentrations and the Michaelis-Menten constants. This means that as [E_a_]_T_ is raised, such that [X](0) decreases and [X^*^](0) increases, initially E_a_ will remain fully saturated, while E_d_ will become saturated. This implies that there is a range of E_a_ concentrations where the influx *J* is still constant but the deactivation rate *γ* is no longer proportional to [X^*^]. In this regime, the concentration of X^*^ increases with a decreasing diffusion constant. Indeed, in this range, where [E_a_]_T_ < [E_d_]_T_, the spatially non-uniform network will respond stronger than the spatially uniform network (see Figures 3 and 5A–5C).

The significance of the diffusion term for [E_d_X^*^] in Equation 14 impedes a transparent analytical derivation of [X^*^](*x*) in this regime (see Text S1). However, under the condition that the influx *J* is constant, we can prove that the total amount of X^*^ must decrease with increasing diffusion constant when *γ* is no longer proportional to [X^*^]. The proof can be found in Text S1.

Here we give a more intuitive explanation for the observation that a network in which the enzymes are spatially separated can respond stronger than a network in which the enzymes are similarly distributed in space. Ultimately, it is a consequence of the nonlinear enzyme–substrate binding curve and the resulting hyperbolic dependence of the deactivation rate *γ* on [X^*^] (see Equation 17). More specifically, this effect can arise when the diffusion constant is low and/or the deactivating enzyme operates close to, but not at, saturation in the uniform system; in this uniform system, X^*^ is distributed evenly through the cell and all particles X^*^ experience the same deactivation rate *μ*. In the spatially non-uniform system, [X^*^] is higher near the pole. If all the deactivating enzyme molecules would operate in the linear regime, i.e., if all deactivating enzyme molecules would not be saturated, then all particles X^*^ would still experience the same degradation rate *μ*; in this scenario, the increase in the number of X^*^ particles close to the pole would precisely balance the decrease in the number of X^*^ particles farther away from the pole, as compared with the uniform network. However, if the concentration of the deactivating enzyme with respect to that of its substrate is lower, i.e., if the enzyme operates close to saturation, then the scenario can arise that the deactivating enzyme molecules near the pole become saturated (Figure 5B), while in the corresponding uniform network they are not. In this scenario, the X^*^ particles that are located close to the pole in the non-uniform network experience a lower effective deactivation rate than the X^*^ particles in the spatially uniform network. This will enhance the response of the non-uniform system as compared with that of the uniform system.

### Strong Activation

We now discuss the effect of the diffusion speed on the concentration profiles of X^*^ when [E_a_]_T_ > [E_d_]_T_ (see Figure 5D–5F). In this regime, [X] is low and [X^*^] is high. This reverses the saturation behavior of the antagonistic enzymes: while in the weak-activation limit E_a_ is saturated and E_d_ is unsaturated, now E_a_ is unsaturated and E_d_ is fully saturated. This also reverses the sensitivities of the influx *J* and the deactivation rate *γ* to changes in the substrate concentration. Indeed, in the strong-activation regime the influx *J* is not constant, but rather the deactivation rate is: *γ = k*_6_[E_d_]_T_ (see Equation 17).

In the limit that the deactivation rate *γ* is constant, Equation 15 can be solved in steady state (see Text S1). The solution is

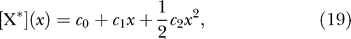
where *c*_2_ = *k*_6_[E_d_]_T_ / *D*, *c*_1_ = *k*_6_[E_d_]_T_ *L* / *D*, and *c*_0_ = [X^*^](0) = [S]_T_ − [E_d_]_T_ (1 + *k*_6_ / *k*_3_(1+ *K*_M,A_ / [E_a_]_T_ )). It is seen that in the high-activation regime, the concentration profile decays algebraically, rather than exponentially, as in the limit of weak activation. This is precisely because in the high-activation regime the total deactivation rate *γ* is constant in space, while in the weak-activation limit *γ* is proportional to the concentration of X^*^, which varies in space. In fact, in the weak-activation limit the deactivation rate *per particle* is constant in space and equal to *μ*. In contrast, in the strong-activation regime, the deactivation rate per particle is not only lower than *μ* on average, but also varies in space: the higher [X^*^] as compared with [E_d_]_T_ (which is constant in space and sets the total deactivation rate), the lower the deactivation rate per particle; activated particles close to the pole thus experience a lower deactivation rate and hence travel farther on average before they are deactivated.

The expression for *c*_0_ = [X^*^](0) reveals that as [E_a_]_T_ increases, the concentration of X^*^ close to the pole where E_a_ is located, increases. In the limit that [E_a_] → ∞, [X^*^](0) → [S]_T_ − 2[Ed]_T_ ≈ [S]_T_, which means that *close* to the pole of the cell where the activating enzyme is located, all the substrate X is converted into X^*^ and E_d_X^*^ (see Figure 5D). Importantly, Figure 5D also shows that as the distance from the pole increases, the fraction [X^*^]_T_(*x*) / [S]_T_(*x*) decreases, *even in the limit that* [E_a_]_T_ ≫ [E_d_]_T_. When [E_a_]_T_ ≫ [E_d_]_T_, all the substrate molecules at the pole will indeed be modified. However, these molecules will then diffuse away from the pole into the cytoplasm, where they can be demodified by the deactivating enzyme molecules, but not remodified. Hence, when the activating enzyme is spatially separated from the deactivating enzyme, it will never be possible to convert all the substrate molecules in the system (see Figure 2). This is in marked contrast with the situation in which the activating and deactivating enzymes are not spatially separated. In this case, all substrate molecules can be converted into X^*^ when [E_a_]_T_ ≫ [E_d_]_T_ (see Figure 2).

The expression for *c*_0_ = [X^*^](0) also reveals that in the limit that [E_a_]_T_ ≫ [E_d_]_T_, the concentration of X^*^ at *x* = 0, is independent of the diffusion constant. However, while [X^*^](0) does not depend on the diffusion constant, the rate at which [X^*^](*x*) decays with the distance from the pole, does depend on it. Equation 19, with *c*_1_ = *k*_6_[E_d_]_T_ *L* / *D*, shows that the concentration profile of X^*^ decays more slowly when the diffusion constant increases (see also Figure 5D). These two observations, when taken together, imply that the total concentration of X^*^ in the whole system increases with increasing diffusion constant. This can be verified by integrating Equation 19 over the length of the cell, which gives [X^*^]^cell^ ∼ *a* − *b* / *D*, where *a* and *b* are positive constants.

These results can be understood by comparing the influx of X^*^ with the efflux of X^*^. When [E_a_]_T_ > [E_d_]_T_, the deactivation rate is constant and hence independent of the diffusion constant. Since the total deactivation rate of X^*^ is independent of the diffusion constant, the total influx of X^*^, which in steady state must balance the total efflux by deactivation, is also independent of the diffusion constant. The influx of X^*^ depends on [E_a_X] and thus on the concentration of X^*^ at *x* = 0, as discussed above. Hence, the concentration of X^*^ at *x* = 0 must be independent of the diffusion constant. A more intuitive explanation is as follows: as the diffusion constant increases, the X^*^ molecules will diffuse away from the pole more rapidly. This would tend to lower the concentration of X^*^ at *x* = 0. However, this process is accompanied by an increase in the flux of X toward the pole ([S]_T_(*x*) is constant); because in the strong-activation limit E_a_ is unsaturated, this would tend to increase [E_a_X] and thereby the influx of X^*^, which would raise the concentration of X^*^. In steady state, these processes balance each other such that the concentration of X^*^ at contact does not depend on the diffusion constant. However, while [X^*^](0) does not change with the diffusion constant, the X^*^ molecules do diffuse away from the pole more rapidly when the diffusion constant increases. This means that the total concentration profile of X^*^ must increase with an increasing diffusion constant (see Figure 3). Indeed, only in the limit that *D* → ∞ and [E_a_]_T_ ≫ [E_d_]_T_, can all the substrate molecules be converted into X^*^ (see Figure 3 and Figure 5D). In the strong-activation limit, spatially separating the antagonistic enzymes thus always weakens the response, in contrast to the behavior in the weak-activation limit.

It should also be noted that the decay length of the concentration profile of X^*^, given by *c*_1_ = *k*_6_[E_d_]_T_ *L* / *D*, does not only depend upon the diffusion constant, but also upon the activity of the deactivating enzyme. In the spatially non-uniform system, the maximum response (i.e., the response when [E_a_]_T_ ≫ [E_d_]_T_) decreases as the catalytic activity of the deactivating enzyme, *k*_6_, increases. The reason is that the X^*^ molecules will travel a shorter distance before they are deactivated, when the deactivation rate is higher. The extent to which spatially separating the enzymes weakens the maximum response thus depends upon both the diffusion constant and the deactivation rate of the X^*^ molecules.

### Space-Dependent Amplification

Figure 6 shows that if the activating enzyme is localized at one pole of the cell, while the deactivating enzyme can freely diffuse through the cytoplasm, the response of the network will depend upon the position in the cell. As can be deduced from Figures 4 and 5, [E_d_X^*^] depends significantly on the position in the cell when [E_a_]_T_ < [E_d_]_T_. When [E_a_]_T_ > [E_d_]_T_, however, [E_d_X^*^] becomes virtually independent of the position *x*, because then all the deactivating enzyme molecules are saturated. The opposite trend is observed for [X^*^]: when [E_a_]_T_ < [E_d_]_T_, [X^*^] is low everywhere in the cell, while if [E_a_]_T_ > [E_d_]_T_, [X^*^] strongly depends upon the position in the cell. The reason is, as discussed in the previous section, that even when [E_a_]_T_ ≫ [E_d_]_T_, not all X can be converted into X^*^ if the two antagonistic enzymes are spatially separated.

Interestingly, the average response of [E_d_X^*^] in the spatially non-uniform system is very similar to that in the system in which the two enzymes are not spatially separated. Yet, the response of [X^*^] does differ markedly between the two systems. This is a result of the strong nonlinearity in the amplification mechanism of zero-order ultrasensitivity: because the activation and deactivation reactions are zero-order in the substrate concentrations [X] and [X^*^], respectively, even when *k*_3_[E_a_X] is only marginally larger than *k*_6_[E_d_X^*^], predominantly all X molecules will be converted into X^*^ [4].

Lastly, Figure 6A shows that the inflection point of the dose–response curve depends on the position *x* in the cell. The inflection point shifts to higher [E_a_]_T_ / [E_d_]_T_ as the distance from the anterior pole increases; this effect becomes more pronounced as *D* decreases (unpublished data). The fact that the inflection point depends on position *x* is one of the principal reasons why the response in the spatially non-uniform system is weaker than that of the uniform system.

### Push–Pull Networks in the Linear Regime

Push–pull networks in living cells are not always in the zero-order regime [4,23]. In the linear regime, push–pull networks do not amplify signals, but can enhance the reliability of cell signaling by making it robust against fluctuations in the concentrations of the components due to noise in gene expression [24]. It is therefore meaningful to study how the input–output relation of a push–pull network in the linear regime depends upon the spatial distribution of the antagonistic enzymes. A push–pull network in the linear regime is given by:

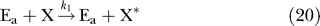


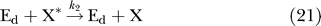



The steady-state concentration profiles for these linear push–pull networks can be derived analytically.

The principal result is that for push–pull networks that are in the linear regime, spatially separating the antagonistic enzymes *always weakens the response*. This can be seen by comparing the response curve for [S]_T_ = 0.4 *K*_M_ in Figure 2A with that in Figure 2B. The reason why for linear networks spatially separating the enzymes reduces the response in the strong-activation limit is the same as that for zero-order networks. The reason that, in contrast to zero-order networks, the response is also weakened in the weak-activation limit, is more subtle. In zero-order networks that are in the weak-activation limit, E_a_ is saturated, and, consequently, the influx *J* is independent of the concentration of X at the pole. In linear networks, E_a_ is unsaturated and the influx *J* is proportional to [X](0). As *D* decreases, [X^*^](0) tends to increase and [X](0) tends to decrease ([S]_T_(*x*) is constant in space). Because in the linear regime *J* is proportional to [X](0), this would lower the influx of X^*^, which, in turn, would lower the concentration of X^*^. Spatially separating the antagonistic enzymes thus amplifies weak signals if the push–pull network operates in the zero-order regime, but not in the linear regime.

## Discussion

In a push–pull network that operates deeply in the zero-order regime, the activation rate is given by *k*_3_[E_a_]_T_, while the deactivation rate is given by *k*_6_[E_d_]_T_; both rates are thus independent of the substrate concentration. If both enzymes are uniformly distributed, or colocalized, then essentially all substrate molecules will be activated when *k*_3_[E_a_]_T_ > *k*_6_[E_d_]_T_, while they will be predominantly deactivated when *k*_3_[E_a_]_T_ < *k*_6_[E_d_]_T_. To drive the modification reactions to completion, it is indeed essential that the antagonistic enzymes are not spatially separated. If the antagonistic enzymes are separated, then the enzyme with the lower global activity can locally still have a higher activity than the other enzyme. More in general, spatially separating the enzymes means that the balance between activation and deactivation depends upon the position in the cell, and this “smearing” of the response always tends to reduce the sharpness of the global response curve.

If information about changes in the environment has to be transmitted, then the gain—the change in the output divided by the change in the input—is a critical quantity. In fact, the *maximum* gain is then usually the most relevant quantity, because signaling networks are often tuned to this point of maximum gain: the input–output function of a module and the concentration of its input signal are often optimized with respect to each other. The intracellular chemotaxis network of E. coli provides a clear example: the steady-state intracellular concentration of the messenger CheYp is around 3 *μ*M, which is precisely the concentration at which the flagellar motors respond most strongly. Our analysis shows that from the perspective of signal amplification, the best strategy is to either colocalize the antagonistic enzymes or to uniformly distribute them in space: spatially separating the enzymes always weakens the maximum response.

Nevertheless, as mentioned in the Introduction, spatial gradients of messenger proteins are often observed. Indeed, maximizing the gain is not the only design principle in cell signaling. Firstly, while in some cases, such as E. coli chemotaxis, the signal has to be transmitted to a large number of places throughout the cell's cytoplasm or membrane [12], in other cases the signal has to be transmitted to distinct regions, such as the nucleus, or be confined to a small region near the membrane, as in the yeast pheromone response where the shmoo tip has to be formed locally; in this scenario, spatial gradients might be important, since they allow the cell to confine signaling to a narrow domain below the cell membrane [9,13]. Secondly, a sharp response may not always be desirable. In order to respond strongly to changes in the input signal over a broad range of input signal strengths, the cell does not only need a sharp response curve, but it also needs to develop elaborate adaptation mechanisms that can reset the network to the point of maximum gain. In E. coli, for instance, the methylation and demethylation enzymes CheR and CheB continually adjust the activity of the receptor cluster, such that the steady-state intracellular CheYp concentration is at 3 *μ*M. A weaker response curve, however, would allow the cell to have a reasonable working range without adaptation mechanisms. In this scenario, not only the maximum gain would be important, but, in fact, the full response curve. Thirdly, it might not always be possible to maximize signal amplification by optimizing the input–output function of a module with respect to its incoming signal, because, for instance, the downstream module also has to respond to other incoming signals, while the signal also has to act on other downstream modules; the yeast MAPK (mitogen-activated protein kinase) pathways, which exhibits cross-talk, provides a prominent example of such a scenario. It seems likely that in this case the full response curve, with the absolute concentrations of the components, is important. In this context, it is interesting to note that spatially separating the antagonistic enzymes weakens strong signals by reducing the maximum output signal (Figures 3 and 5A), while it can enhance weak signals if the network operates in the zero-order regime (Figures 3 and 5D). This dependence of the input–output relation on the spatial distribution of the antagonistic enzymes could be exploited by cells to relay different environmental signals specifically.

The analysis performed here is essentially a mean-field analysis. It is assumed that the concentrations are large and that fluctuations can be neglected. However, in the living cell, the concentrations are often low, which means that fluctuations can be important. This is particularly relevant for push–pull networks. Their high gain not only amplifies the mean of the input signal, but will also amplify the noise in the input signal [8,25,26]. Moreover, when the modification reactions become more zero-order, the intrinsic fluctuations of the push–pull network, i.e., noise resulting from the modification reactions themselves, will also increase [5]. In fact, it has been shown that when push–pull networks operate deeply in the zero-order regime, fluctuations can lead to a bimodal response [7]. All these analyses of the effect of noise on the amplification mechanism of zero-order ultrasensitivity have been performed under the assumption that the enzymes are uniformly distributed in the cytoplasm. It would clearly be of interest to study the effect of enzyme (co)-localization on the noise characteristics of push–pull networks.

Finally, could our predictions be tested experimentally? To test our predictions, one would ideally like to perform an experiment on a system with a canonical push–pull network in which all the parameters—concentrations of components, rate constants, diffusion constants—are kept constant, except for the spatial location of one of the enzymes. This clearly seems a very difficult experiment to perform, and to our knowledge, no such experiment has been performed yet, with the possible exception of the experiment by Vaknin and Berg [10]. Vaknin and Berg studied the effect of phosphatase localization on the response of the intracellular chemotaxis network of E. coli cells. This network has a topology that is very similar to that of the canonical push–pull networks considered here, and it is believed that in the wild-type cells both the kinase and the phosphatase are localized at the cell pole. Vaknin and Berg compared the response of wild-type cells to that of mutant cells, in which the phosphatase was mutated such that it freely diffuses in the cytoplasm. They found that the spatial distribution of the phosphatase can have a marked effect on the sharpness of the response, which seems to support the principal conclusion of our analysis. We would like to emphasize, however, that to assess the importance of the spatial distribution of the antagonistic enzymes in a push–pull network, a careful, quantitative analysis of the network is required. First of all, our analysis shows that both the quantitative and qualitative consequences of enzyme localization depend upon the regime in which the network operates. For instance, our calculations reveal that if the activation rate is independent of the messenger concentration, and if the deactivation rate is linear in the messenger concentration, then the localization of the phosphatase should have no effect at all on the response curve. Secondly, it is quite possible that in the mutant cells not only the spatial distribution of the enzymes is different, but also their expression levels, and even other parameters such as rate constants. In fact, experiments by Wang and Matsumura suggest that the activity of the phosphatase in the E. coli chemotaxis network is enhanced at the receptor cluster [27]. Clearly, different rate constants would also tend to change the response curve of the mutant cells with respect to that of the wild-type cells. To elucidate the effect of enzyme localization on the dose–response curve of a network thus requires quantitative experiments and quantitative modeling. In a future publication, we will present a detailed analysis on the importance of phosphatase localization in the chemotaxis network of E. coli.

## Supporting Information

Text S1 Enzyme Localization Can Drastically Affect Signal Amplification in Signal Transduction Pathways (260 KB DOC)
