# Supplementary material for: Enzyme Localization Can Drastically Affect Signal Amplification in Signal Transduction Pathways
Source: PLoS Comput Biol. 2007 Oct 12;3(10):e195. doi: 10.1371/journal.pcbi.0030195 (PMC2014792; doi:10.1371/journal.pcbi.0030195)
Supplement: Supplementary file 1 [file pcbi.0030195.sd001.doc]

**Supporting Text**

***Enzyme localization can drastically affect signal amplification in signal transduction pathways***

Siebe B. van Albada and Pieter Rein ten Wolde
*FOM Institute for Atomic and Molecular Physics,*

*Kruislaan 407, 1098 SJ Amsterdam, The Netherlands.*

The spatio-temporal evolution of the concentration profiles of a push-pull network where the activating enzyme is located at one pole of the cell, while the deactivating enzyme freely diffuses through the cytoplasm, is given by Eqs. 9-14 of the main text. To derive the spatio-temporal evolution of $[\text{X}^{*}]$ as given by Eqs. 15-17 of the main text, we have to make the assumptions that the formation of the enzyme-substrate complexes is fast and that the diffusion term for $[\text{E}_{\text{d}}\text{X}^{*}]$ in Eq. 14 can be neglected. The steady-state solutions of Eqs. 12 and 14 can then be combined with Eq. 9 to yield Eq. 15 of the main text. We now discuss the steady-state solution of Eq. 15 in the limits of weak-activation and strong-activation separately.

**Weak activation**

In the limit that $[\text{E}_{\text{a}}]_{\text{T}}\ll[\text{E}_{\text{d}}]_{\text{T}}$, the diffusion term in Eq. 14 of the main text will be small compared to the other terms in steady-state. Combining the steady-state solution of that equation with those of Eqs. 9 and 12 of the main text, yields the following equation for $\text{X}^{*}$ in steady state:

$D\frac{\text{d}^{2}[\text{X}^{*}]}{\text{d}x^{2}} =-k_{3}[\text{E}_{\text{a}}]_{\text{T}}\delta(x)\frac{[\text{X}](0)}{K_{\text{M},\text{a}}+[\text{X}](0)}$ (1)

$+k_{6}[\text{E}_{\text{d}}]_{\text{T}}\frac{[\text{X}^{*}]}{K_{\text{M},\text{d}}+[\text{X}^{*}]}$ (2)

In the limit that $[\text{E}_{\text{a}}]_{\text{T}}\ll[\text{E}_{\text{d}}]_{\text{T}}$, $[\text{X}]$ will be large and $[\text{X}^{*}]$ will be small. In this limit, the above equation reduces to

$D\frac{\text{d}^{2}[\text{X}^{*}]}{\text{d}x^{2}}\approx-k_{3}[\text{E}_{\text{a}}]_{\text{T}}\delta(x)+k_{6}/K_{\text{M},\text{d}}[\text{E}_{\text{d}}]_{\text{T}}[\text{X}^{*}].$ (3)

This equation can be solved with the boundary conditions

$-D\left.\frac{\text{d}[\text{X}^{*}](\text{x})}{\text{d}x}\right|_{x=0} =J, \left.\frac{\text{d}[\text{X}^{*}](x)}{\text{d}x}\right|_{x=L} =0,$ (4)

where $J=k_{3}[\text{E}_{\text{a}}]_{\text{T}}$ and $L$ is the length of the cell. Defining the effective deactivation rate of $\text{X}^{*}$ to be $\mu=k_{6}/K_{\text{M},\text{d}}[\text{E}_{\text{d}}]_{\text{T}}$ and the characteristic decay length of $\text{X}^{*}$ to be $\lambda=\sqrt{D/\mu}$, then, if $L\gg\lambda$, the solution of the above equation is

$[\text{X}^{*}](x)=\frac{J\lambda}{D}\exp(-x/\lambda).$ (5)

This equation predicts that the total amount of $\text{X}^{*}$ in the whole cell is independent of the diffusion constant $D$. This equation holds only in the limit that the diffusion term for $[\text{E}_{\text{d}}\text{X}^{*}]$ in Eq. 14 of the main text can be neglected and if the deactivating enzyme $\text{E}_{\text{d}}$ is unsaturated, meaning that the total deactivation rate $\gamma$ is proportional to the concentration of $\text{X}^{*}$. These assumptions are only accurate when $[\text{E}_{\text{a}}]_{\text{T}}\ll[\text{E}_{\text{d}}]_{\text{T}}$.

As $[\text{E}_{\text{a}}]_{\text{T}}$ increases, $[\text{X}^{*}]$ increases, and close to the pole where the activating enzyme resides, the deactivating enzyme does become saturated with $\text{X}^{*}$. This lowers the deactivation rate *per particle*, which in turn increases the concentration of $\text{X}^{*}$ with respect to that in a system where both enzymes are uniformly distributed in space.

The significance of the diffusion term for $[\text{E}_{\text{d}}\text{X}^{*}]$ impedes an analytical derivation of $[\text{X}^{*}](x)$. However, when $[\text{E}_{\text{a}}]_{\text{T}}<[\text{E}_{\text{d}}]_{\text{T}}$ and $[\text{X}]$ is large, $[\text{E}_{\text{a}}\text{X}]$ is essentially constant and independent of the diffusion constant. Under this condition we can prove that the total amount of $\text{X}^{*}$ must decrease with increasing diffusion constant, implying that in this regime the uniform network will actually respond weaker than the spatially non-uniform network. Integrating Eq. 14 over the length of the cell reveals that in steady state $k_{4}\int_{0}^{L} dx[\text{E}_{\text{d}}](x)[\text{X}^{*}](x)=(k_{5}+k_{6})\int_{0}^{L} dx[\text{E}_{\text{d}}\text{X}^{*}](x)$. Combining Eq. 9 and Eq. 14 reveals that in steady state $k_{3}[\text{E}_{\text{a}}\text{X}]=k_{6}\int_{0}^{L} dx[\text{E}_{\text{d}}\text{X}^{*}](x)$. Hence, in steady state $[\text{E}_{\text{a}}\text{X}]=c\int_{0}^{L} dx[\text{E}_{\text{d}}](x)[\text{X}^{*}](x)$, where $c$ is a constant. This means that if $[\text{E}_{\text{a}}\text{X}]$ is independent of the diffusion constant, also $\int_{0}^{L} dx[\text{E}_{\text{d}}](x)[\text{X}^{*}](x)$ must be independent of the diffusion constant. Now, when the diffusion constant decreases, the profiles $[\text{E}_{\text{d}}](x)$ and $[\text{X}^{*}](x)$ vary more strongly in space and become less overlapping, as Figs. 5a and 5c show. The decrease in overlap between $[\text{E}_{\text{a}}](x)$ and $[\text{X}^{*}](x)$ would tend to decrease $\int_{0}^{L} dx[\text{E}_{\text{d}}](x)[\text{X}^{*}](x)$. To compensate for this, the total amount of $\text{X}^{*}$ must increase with decreasing diffusion constant, when $[\text{E}_{\text{a}}]_{\text{T}}<[\text{E}_{\text{d}}]_{\text{T}}$ and $[\text{E}_{\text{a}}\text{X}]$ is constant.

**Strong activation**

Combining Eq. 9 with Eq. 14 yields, in steady state:

$D\frac{\text{d}^{2}[\text{X}^{*}](x)}{\text{d}x^{2}}=-k_{3}[\text{E}_{\text{a}}\text{X}]\delta(x)-D\frac{\text{d}^{2}[\text{E}_{\text{d}}\text{X}^{*}](x)}{\text{d}x^{2}}+k_{6}[\text{E}_{\text{d}}\text{X}^{*}](x).$ (6)

This equation for the steady-state concentration profile of $\text{X}^{*}$ is exact. Nevertheless, the diffusion term for $[\text{E}_{\text{d}}\text{X}^{*}]$ impedes a transparant analytical solution. However, if $[\text{E}_{\text{a}}]_{\text{T}}>[\text{E}_{\text{d}}]_{\text{T}}$ and $D$ is not too low, then essentially all of the deactivating enzyme is saturated, and $[\text{E}_{\text{d}}\text{X}^{*}](x)=[\text{E}_{\text{d}}]_{\text{T}}(x)\equiv[\text{E}_{\text{d}}](x)+[\text{E}_{\text{d}}\text{X}^{*}](x)$ (see Fig.5e of main text). Moreover, combining Eqs. 13 and 14 of the main text reveals that $[\text{E}_{\text{d}}]_{\text{T}}(x)$ is constant in space, if, as assumed here, the diffusion constants of the enzyme $\text{E}_{\text{d}}$, and that of the enzyme bound to its substrate, $\text{E}_{\text{d}}\text{X}^{*}$, are the same. Hence, in the limit that $[\text{E}_{\text{a}}]$ is high and $\text{E}_{\text{d}}$ is saturated, the second term on the right-hand side of the above equation is zero, the third term is constant, and the equation reduces to

$D\frac{\text{d}^{2}[\text{X}^{*}](x)}{\text{d}x^{2}}=-k_{3}[\text{E}_{\text{a}}\text{X}]\delta(x)+k_{6}[\text{E}_{\text{d}}]_{\text{T}}.$ (7)

This equation can be solved with the boundary conditions in Eq. 4 with $J=k_{3}[\text{E}_{\text{a}}\text{X}]$. The solution is

$[\text{X}^{*}](x)=c_{0}+c_{1}x+\frac{1}{2}c_{2}x^{2},$ (8)

where $c_{2}=k_{6}[\text{E}_{\text{d}}]_{\text{T}}/D$, obtained from the solution of Eq. 7 in the domain $0<x<L$, and $c_{1}=-k_{6}[\text{E}_{\text{d}}]_{\text{T}}L/D$, obtained from the boundary condition at $x=L$. The coefficient $c_{0}=[\text{X}^{*}](0)$ can be obtained from the boundary condition at $x=0$: $k_{6}[\text{E}_{\text{d}}]_{\text{T}}L=k_{3}[\text{E}_{\text{a}}\text{X}]$. First, we note that the total substrate concentration in the *cytoplasm*, $[\text{S}]_{\text{c}}(x)\equiv[\text{X}](x)+[\text{X}^{*}](x)+[\text{E}_{\text{d}}\text{X}^{*}](x)=[\text{S}]_{\text{c}}$, is constant in space, because the diffusion constants of all the diffusing components are equal. Hence, the total substrate concentration in the whole cell is $[\text{S}]_{\text{T}}\equiv[\text{S}]_{\text{c}}+[\text{E}_{\text{a}}\text{X}]/L=[\text{S}]_{\text{T}}=[\text{X}](x)+[\text{E}_{\text{a}}\text{X}]/L+[\text{X}^{*}](x)+[\text{E}_{\text{d}}]_{\text{T}}$ (where we have used that $[\text{E}_{\text{d}}\text{X}^{*}](x)\approx[\text{E}_{\text{d}}]_{\text{T}}$). The concentration $[\text{X}](0)$ can be obtained by solving Eq. 12 of the main text in steady state, which gives $[\text{X}](0)=K_{\text{M},\text{a}}[\text{E}_{\text{a}}\text{X}]/(L[\text{E}_{\text{a}}]_{\text{T}}-[\text{E}_{\text{a}}\text{X}])$. Combining these expressions with the boundary condition at $x=0$, and using that when $[\text{E}_{\text{a}}]_{\text{T}}$ is high, $[\text{E}_{\text{a}}]_{\text{T}}\gg[\text{E}_{\text{a}}\text{X}]$, yields $c_{0}=[\text{X}^{*}](0)=[\text{S}]_{\text{T}}-[\text{E}_{\text{d}}]_{\text{T}}(1+k_{6}/k_{3}(1+K_{\text{M},\text{a}}/[\text{E}_{\text{a}}]_{\text{T}}))$.
